# Sexual Knowledge, attitudes and behaviors among unmarried migrant female workers in China: a comparative analysis

**DOI:** 10.1186/1471-2458-11-917

**Published:** 2011-12-12

**Authors:** Jie Tang, Xiaohui Gao, Yizhen Yu, Niman Isse Ahmed, Huiping Zhu, Jiaji Wang, Yukai Du

**Affiliations:** 1 Department of Child, Adolescence & Woman Health Care, School of Public Health, Tongji Medical College, Huazhong University of Science & Technology, 13th Hangkong Road, Wuhan 430030, P.R. China; 2 Guangzhou Medical College, 195th Dongfengxi Road, Guangzhou 510182, P.R China

## Abstract

**Background:**

In recent years, many studies have focused on adolescent's sex-related issues in China. However, there have been few studies of unmarried migrant females' sexual knowledge, attitudes and behaviors, which is important for sexual health education and promotion.

**Methods:**

A sample of 5156 unmarried migrant female workers was selected from three manufacturing factories, two located in Shenzhen and one in Guangzhou, China. Demographic data, sexual knowledge, attitudes and behaviors were assessed by self-administered questionnaires. Multivariate logistic regression analysis was conducted to examine the factors associated with premarital sexual intercourse.

**Results:**

The average age of the unmarried female workers included in the sample was 20.2 years, and majority of them showed a low level of sex-related knowledge. Females from the west of China demonstrated a significant lower level of sex-related knowledge than those from the eastern or central provinces (*p *< 0.05). Approximately 13% of participants held a favorable attitude towards premarital sexual intercourse, and youths from the east/central were more likely to have favorable attitudes compared with those from the west (*p *< 0.05). About 17.0% of the unmarried female workers reported having engaged in premarital sexual intercourse, and females from the east/central were more likely to have experienced premarital sexual intercourse than those from the west (*p *< 0.05). Multivariate analysis revealed that age, education, current residential type, dating, sexual knowledge, attitudes, and pattern of communication were significantly associated with premarital sexual intercourse.

**Conclusion:**

The unmarried migrant female workers lack sexual knowledge and a substantial proportion of them are engaged in premarital sexual behaviors. Interventions aimed at improving their sexual knowledge and related skills are needed.

## Background

Health risk behaviors such as cigarette smoking, weapon-carrying, and unprotected sexual intercourse contribute to the leading causes of morbidity, mortality, and social problems among adolescents and youths [1]. Premarital sex among adolescents and youths predispose them to unwanted pregnancies, unsafe abortions, pregnancy-related complications, and sexually transmitted infectious (STI) including HIV/AIDS. The United Nations estimated that about half of new HIV infectious worldwide occur among young people aged 15~24 years [2]. In China, more than 60% of all HIV infectious are among youth [3]. A systematic review of surveys from five provinces in China showed over 54% of urban women interviewed prior to marriage had experienced sexual intercourse, up to one third had been pregnant, and almost all of these had an induced abortion [4].

Under the influence of mass media, rapid modernization, economic expansion, and exposure to new ideas, sexual attitudes and norms have been changing among adolescents and young people in developing countries, including China. Emerging evidence from rural and urban areas shows that premarital sex is no longer taboo [5], and that there is an increase in premarital sexual activity among adolescents and young people in China [6,7]. A study conducted in 2001 reported that 43% of boys and 25% of girls said yes to premarital sex, a significant increase from 34% and 13%, respectively, since 1995 [8]. The rapid economic development has brought about increase in number of migrants who migrate to areas with greater economic opportunities. In China, it is reported that the total number of migrants had reached 0.15 million in 2005, and a large proportion of them were unmarried [9].

Being away from home is associated with an increase in risk behaviors in both developed and developing countries [10,11]. Young migrant workers are more likely to encounter problems as a result of broad changes in lifestyle combined with socio-economic and cultural shock, which may have an impact on their attitude towards marriage and sexual life [12]. For example, Zhang et al reported that 12.6% of unmarried female migrant workers had sex experiences and 29.9% of them had at least one induced abortion [13]. The secular trend in puberty led to the onset of menarche 2~3 years earlier over the past century, which may contribute to an earlier initiation sexual activity. A study conducted in Taiwan found that the average age of the first sexual experience for boys was 16.1 years old, which was earlier than ten years ago [8].

Unmarried migrant youths are even more vulnerable to having premarital sexual intercourse. Most of them lack knowledge and skills to avoid risky behaviors, such as unprotected sex, and are unable to access or are restricted from receiving affordable and appropriate reproductive health information and services [14,15]. Premarital sexual activity is more likely to put the unmarried youth at risk of unwanted pregnancy, unsafe abortion, sexually transmitted infectious (STI) and HIV/AIDS [16].

The most powerful influences on human sexuality are the social norms that govern its expression [17]. Morality, taboos, laws, and religious beliefs are used by societies worldwide to circumscribe and radically determine the sexual behavior of their citizens. Furthermore, the regional variation in sexual behavior underlines the powerful role of environment factors in shaping behavior and its consequences for sexual health [16]. Migrants from the western and east/central provinces of China are different in many aspects, including social-economic status, culture and religious beliefs, all of which may bring different attitudes towards sexual health. However, few studies have explored the regional differences of sexual attitudes and behavior among unmarried migrant female in the Chinese context.

The present study was designed to address three primary research questions: (a) what are the regional differences on sexual attitudes and the level of sex-related knowledge among unmarried female migrant workers? (b) How prevalence is premarital sexual intercourse among unmarried female migrant workers and the regional differences? (c) What are the factors that are associated with premarital sexual intercourse among unmarried female migrant workers and the regional differences? This information is important for policy makers and heath educators to develop effective and feasible strategies targeting at promoting sexual health.

## Methods

### Study sites and participants

The study was carried out from July 2009 to December 2010 in Guangzhou and Shenzhen, two large cities in Guangdong province located in the south of China, a highly industrialized province. The local Central Diseases Control Agency helped to locate three factories, two in Shenzhen and one in Guangzhou which employed many young migrants, and where the managers were willing to cooperate with this study. All of these factories are Taiwanese owned and assemble electronic products (mobile phones, computers, etc). There are about 20, 000 employees in each factory, 65% are female, they work 48 hours a week and their monthly salary ranges from 1000 to 2000 RMB (Renminbi) which is approximately equivalent to 150 to 300 USD. To assure adequate proportions of female workers across the regions (the east/central and the west of China), sampling on each factory was purposive. Because few female workers were from the west of China, we endeavored to recruit female higher proportion from the west. To be eligible for this study, female workers needed to be unmarried and less than one year since leaving their home town. The research protocol, including the questionnaires, was approved by the three factories and the Ethics Committee of the Medical Association of Tongji Medical College of Huazhong University of Science and Technology. We obtained written informed consent from each participant. Initially, a total sample of 5534 migrant female workers were obtained, however, 173 subjects who had left their home town more than 1 year previously and 205 subjects who were married were excluded. The final study population consisted of 5156, 93.2% of those initially recruited with a mean age of 20.2 years (SD = 2.2).

The survey was completed in a private environment (e.g., dining room of the factories). All the participants were required not to identify themselves by writing their names on the questionnaire and participants were assured of the confidentiality of their responses. A group of trained investigators provided explanations and instructions for completing the survey, and were available to assist participants with problems in understanding the questionnaire. All the questionnaires were reviewed by the investigators for completeness and consistency.

### Measures

The questionnaire was initially designed in Chinese basing on similar surveys that have been carried out in China [18-20]. It collected information on participants' age, education, home town (the east/central or the west), residential type, and period of time since they left their home, dating status, communication on sex related matters, attitudes toward premarital sex and induced abortion and premarital sexual activities. The questionnaire also contained items regarding sexual physiology, contraceptive methods, and HIV/STI transmission, symptoms, and prevention knowledge. The questionnaire had both closed and open-ended questions and the open-ended answers were coded before analysis. The questionnaire was pre-tested among 75 female migrant workers to ensure content and language were appropriate for the study population. Additionally, test-retest reliability was examined. Participants were surveyed at two time points, seven days apart, with the participants using self-assigned identification numbers so surveys could be linked across time points. A total of 71 pairs of completed questionnaires were obtained. No significant difference over time in proportions reporting premarital sex (χ^2 ^= 0.05, *p *= 0.82), and sexual attitude (χ^2 ^= 0.22, *p *= 0.64), and the knowledge scores across the two time points were highly correlated (r = 0.81, *p *< 0.01).

The primary variables of interest were sex-related knowledge, attitude toward premarital sex induced abortion, and participants' experiences of premarital sex and pregnancy. Premarital sex was indicated by a dichotomous variable: yes or no, and pregnancy involvement denoted whether or not an unmarried migrant female worker had conceived.

Thirty-six items on the questionnaire addressed knowledge of sexual physiology, contraceptive methods, and HIV/STI transmission and prevention. All those questions were coded as a dichotomous variable (false/true) with score ranging from 0 to 1. Three composite scores were created by adding the number of correct answers to 12 questions on sexual physiology, 10 questions on contraceptive methods, and 14 questions on HIV/STI transmission and prevention, and the total score ranged from 0 to 36, with higher scores reflecting increased sex-related knowledge. The consistency as reflected by Cronbach's alpha coefficient of these thirty-six items in our study sample was 0.75.

Each subject was asked "how do you think about having sexual intercourse before marriage?" to measure their attitudes towards premarital sex with five options: (1) it is all right if both of the people are willing to; (2) it is ok only if they have fallen in love; (3) it is a bad thing but I can accept it; (4) I oppose it; (5) the people who have sexual intercourse should be punished by the law. For the purposes of statistical analysis, responses were grouped into 3 categories: approve (option 1 and 2), neutral (option 3), and disapprove (option 4 and 5).

The original question for attitude about premarital pregnancy was "how do you think about having a premarital pregnancy?" Three options were included: (1) it is a bad thing that I can not accept it; (2) it doesn't matter; (3) I am in favor of it. People who selected option 1 were classified as 'disapprove', option 2 as 'neutral', and option 3 as 'approve'.

Communication with others on sexual related matters was measured using the following five questions "(1) have you ever talked about sexual related questions with your parents?", "(2) have you ever talked about sexual related questions with your teacher?", "(3) have you ever talked about sexual related issues with your boyfriend?", and "(4) have you ever talked about sexual related questions with medical staffs?". All of those questions were indicated by a dichotomous variable: yes or no.

### Statistical Analysis

Descriptive statistics (mean, standard deviation, and percentage) were calculated on the demographic characteristics of the sample. Chi-square test and Student *t*-test were used to compare frequencies and continuous data, respectively. Additionally, binary logistic regression analyses were performed to indentify factors associated with premarital sexual intercourse. Significance level was set at 0.05. Statistical analyses were conducted using Statistical Package for Social Sciences software (SPSS for Windows 15.0, SPSS Inc., Chicago, IL).

## Results

### Sample characteristics

The general characteristics of the unmarried female workers are summarized in Table 1. A total of 5156 female unmarried workers (4379 were from eastern/central provinces and 777 were from western provinces) from the three factories were enrolled in this study. There were no significant differences in mean age, education level, residential type or dating status between the eastern/central and the western females (*p >*0.05).

**Table 1:** Descriptive characteristics of the study population

	Eastern/central areas (n = 4379)	Western areas (n = 777)	All (n = 5156)
Age(Mean ± SD)#	20.20 ± 2.18	20.16 ± 2.25	20.19 ± 2.19
Nationality Han (vs. minority) (%)^#^	96.10	96.40	96.2
Education level^#^			
Junior high or lower	56.32	54.41	56.10
Senior high	41.44	43.14	41.70
College or higher	2.24	2.45	2.20
Current residential type^#^			
Rental	10.53	11.11	10.62
Dormitory	89.47	88.89	89.38
Dating^#^	42.09	41.37	42.02

^#^*p *> 0.05

### Sex-related knowledge and attitudes towards premarital sex and pregnancy

The scores of sex-related knowledge are in Table 2. In general, the unmarried female workers had a low level of sex-related knowledge. On average, the subjects obtained a score of 16.65, with 7.2 for sexual physiology, 3.5 for contraceptive methods, and 5.9 for HIV/STI transmission and prevention. The participants with a higher educational level had a significantly higher level of sex-related knowledge (p < 0.05). Subjects from the west of China, had a lower level of knowledge than those from the east/central of China in sexual physiology, contraceptive methods, and HIV/STI transmission and prevention (*p *< 0.001).

**Table 2:** Comparison of sex-related knowledge and attitudes towards premarital sex and pregnancy among unmarried female workers from the Eastern/Central and the Western of China

	Eastern/central (N = 4379)	Western (N = 777)	*t*/χ^2^	*p*
Sex-related knowledge (Mean, SD)				
Sexual physiology	7.34 ± 1.78	6.45 ± 1.68	13.33	0.000
Contraception methods	3.65 ± 1.80	2.79 ± 1.83	12.14	0.000
HIV/STI transmission	6.08 ± 2.22	5.01 ± 2.66	10.69	0.000
Total scores	17.07 ± 4.28	14.25 ± 4.55	16.09	0.000
Attitudes towards premarital sex (N, %)				
Favorable	606 (13.9)	72 (9.3)		
Neutral	634 (14.5)	105 (13.5)	13.81	0.001
Unfavorable	3129 (71.6)	599 (77.2)		
Attitudes towards pregnancy (N, %)				
Favorable	359 (8.2)	43 (5.5)		
Neutral	715 (16.3)	73 (9.4)	34.66	0.000
Unfavorable	3305 (75.5)	661 (85.1)		

A majority (72.3%) of the all participants disapproved of premarital sex, and only 13.1% of participants held a favorable attitude towards premarital sex, 13.9% participants from the east/central compared to 9.3% participants from the west held favorable attitudes towards premarital sex, and significance were found between them (*p *= 0.001). There were 7.8% of the participants who approve of an unmarried pregnancy, and 72.3% of the participants who disapprove of an unmarried pregnancy. Subjects from the west of China were likely to hold unfavorable attitudes towards pregnancy than those from the east/central of China, and significant differences were found (*p *= 0.000).

### Premarital sexual intercourse and pregnancy rate

Overall 17.0% of the subjects reported having engaged in premarital sexual intercourse, and 26.4% of those who had experienced sexual intercourse reported becoming pregnancy. Comparisons of premarital sexual intercourse and pregnancy rate between youths from the west and the east/central of China are shown in Table 3. Significant differences were found in premarital sexual intercourse (*p *= 0.031), but not in the pregnancy (*p *= 0.764).

**Table 3:** Comparison of premarital sexual intercourse and pregnancy among unmarried female workers from the Eastern/Central and the Western of China

	Eastern/central	Western	χ^2^	*p*
Premarital sexual intercourse (n, %)				
yes	764(17.4)	111(14.3)	4.09	0.031
no	3615(82.6)	666(85.7)		
Premarital pregnancy among female who had reported premarital sexual intercourse (n, %)				
yes	203(26.6)	28(25.2)	0.090	0.764
no	561(73.4)	83(74.8)		

### Factors associated with premarital sexual intercourse

To examine factors associated with premarital sexual intercourse in a multivariate context, binary logistic regressions analysis was performed stratified by region, with 'self-reported premarital sexual intercourse' as the dependent variable. Factors included in the regression were 'sexual knowledge' and 'attitudes towards premarital sex', adjusting for 'communication with others on sexual related matters' and 'demographic characteristics'. The results are shown in Table 4. Among the participants, age, education level, current residential type, dating, sexual-related knowledge, and attitude towards premarital sex were significantly associated with premarital sex. Additionally, having communicated with a boyfriend on sex-related issues was significantly associated with premarital sex in the unmarried female workers from the east/central parts of China.

**Table 4:** Logistic regression models predicting premarital sexual intercourse

	Total subjects		Eastern/central subjects		Western subjects	
	
	OR	95% CI for OR	OR	95% CI for OR	OR	95% CI for OR
Age in years	1.25*	1.20~1.30	1.28*	1.23~1.35	1.17*	1.12~1.22
Education (Senior high or higher vs. Junior high or lower)	1.41*	1.20~1.66	1.48*	1.25~176	1.36*	1.14~1.48
Current residential type (Rental vs. Dormitory)	4.83*	3.84~6.07	5.38*	4.19~6.92	3.05*	1.65~5.65
Dating (yes vs. no)	3.69*	3.05~4.46	3.73*	3.05~4.58	4.21*	2.45~7.21
Sexual-related knowledge	1.08*	1.06~1.10	1.05*	1.02~1.07	1.26*	1.19~1.33
Attitudes towards premarital sex (Unfavorable as reference)						
neutral	0.627*	0.49~0.80	0.50*	0.389~0.65	1.09	0.54~2.19
favorable	2.57*	1.93~3.43	2.14*	11.57~2.92	2.45*	1.03~5.79
Communicated with a boyfriend about sex-related issues(yes vs. no)	1.68*	1.34~2.09	1.78*	1.40~2.26	1.50	0.60~3.75
Constant	-9.01		-8.98		-6.64	

CI = Confidence Interval. *p < 0.05, n = 5156.

## Discussion

In this study, we investigated sexual attitudes and behavior among unmarried female factory workers in China. The results revealed that a majority of unmarried female workers in our study disapproved of premarital sex, and only 13.1% of subjects held a favorable attitude towards premarital sex, which is inconsistent with previous studies conducted in China [21,22]. Significant differences on attitudes toward premarital sexual intercourse and pregnancy were found between the west and the east/center provinces, which may be due to differences in social-economic and cultural backgrounds [23].

Over the last two decades, China has experienced profound social changes associated with the economic reforms. Dramatic changes also seem to have occurred in cultural beliefs and behaviors, such as beliefs about sex, premarital sexual intercourse and premarital pregnancy [23]. In our study, the self-reported rate of premarital sexual intercourse was 17.0%, and 26.4% of those who experienced sexual intercourse reported becoming pregnancy. A study conducted in China reported that the rate of premarital sexual intercourse in unmarried migrant females was 26.9% [24], which was higher than in our study. Differences in demographic characteristics may be at least a partial explanation for the study outcomes. In our study, all participants had been away from their home towns for less than one year, and this had been indicated as one of potential risk factors of engaging in premarital sexual intercourse in previous study [25,26]. Additionally, rates of sexual experience reported by females from the east were higher than that reported by females from the west, although they were similar in some demographic characteristics, such as age, education level, dating status, etc. These findings seem to suggest that different social environment promote different attitudes towards premarital sex which may lead to a different sex-related behavior [27,28].

This study showed a low level of sexual knowledge scores for females from both the west and the eastern/central provinces of China. A very small proportion of participants correctly answered all questions. This may indicate that health education in China provides little information on sexual issues to unmarried females [29]. This study also highlights the importance of sex education by showing that the higher the educational level of the participants, the better the understanding of sexual knowledge. This confirms that schools are one of the main sources of knowledge and influence, as well as parents and peer groups. While much effort and many resources have been earmarked for sex education in schools, family education seems to have been ignored, although it is one of the most important parts of education [30].

In our study, the results show that age, education level, current residential type, dating status, sexual-related knowledge, attitudes, and communication with their boyfriend on sex-related issues are associated with premarital sexual intercourse, which are similar to the findings of previous studies [24,31]. Previous studies have suggested that premarital sexual intercourse is a behavior resulting from the interaction of family, social, personal and environmental factors [32,33]. Different types of family environments are postulated to be related to premarital sexual intercourse, such as parents' disciplinary style, parents' marital status, communication status, and even the economic level [22]. Other social and environmental risk factors included occupation, laws, morality, and customs [34]. Personal risk factors include physiological drive, sex-related knowledge, attitude and beliefs [35]. The risk factors can also be classified into three categories: predisposing factors, enabling factors and reinforcing factors. Many researches have found that attitudes to sex have an enormous influence on sexual activities [22,36]. In the present study, current residential type was a prominent factor associated with premarital sex which was consistent with a previous study conducted in China [27],

Our study indicated that females who communicate with boyfriends on sex-related issues were more likely to engage in premarital sex, which was inconsistently reported in previous studies. For example, some previous studies demonstrated a prospective relationship between parent-youth communication and premarital sexual behavior [36]. However, Mallika A et al reported that the exposure to alcohol, drugs or pornographic films and having more frequent interaction with peers were positively associated with romantic and sexual relationships for both young women and young men [37], similar results were also reported by Mee-Lian Wong et al [38]. It should also be noted that the rates of sexual intercourse in this population are far below reported rates for western populations of similar age [39].

The present study must be interpreted in light of several limitations. First, although the study achieved a relative large sample size, we did not use a random sampling method to select the study population, which might have led to selection bias. Some unmarried female workers who had not experienced premarital sexual intercourse might have been more willing to take part in the present study rather than those who were more experienced. However, this is not likely in view of the association between sexual activity and communication shown in this study.

Second, it is possible that the study population underreported their behaviors since sexual behavior is a sensitive subject and socially unacceptable under Chinese culture [40]. However, by ensuring privacy during the completion of questionnaire and using the anonymous self-administered survey, an attempt was made to minimize this bias [41].

Third, we did not consider some other factors which may be associated with the occurrence of premarital sexual intercourse, including parents' occupation, education level, and relationship with their parents. This might/could lead to different models predicting premarital sexual intercourse. Therefore, the investigation of these and other factors remains key directions for future research.

Fourth, this study was a cross-sectional design, and determining causality must be inferred and can not be tested in the data. Therefore, the findings should be validated in future longitudinal research.

## Conclusion

This is the first study that examines sexual knowledge, attitudes, and behaviors among unmarried migrant females in China. The results shows that the unmarried migrant females lack sexual knowledge and a substantial proportion of them are engaged in premarital sexual behaviors. Hence, different interventions that target at improving their sexual knowledge and related skills are needed. Our findings may provide some critical information for sexual health education and promotion.

## Competing interests

The authors declare that they have no competing interests.

## Authors' contributions

DY and WJ participated in the design of the study and performed the statistical analysis. TJ and NIA drafted the manuscript, ZH and GX participated in data collection. YY participated in its design and coordination and helped to draft the manuscript. All authors read and approved the final manuscript.

## Pre-publication history

The pre-publication history for this paper can be accessed here:

http://www.biomedcentral.com/1471-2458/11/917/prepub
